# Supplementary material for: Identification of Freezing-Responsive microRNAs and Their Targets in Chinese Jujube by Small RNA and Degradome Sequencing
Source: Int J Mol Sci. 2026 Sep 4;27(17):7912. doi: 10.3390/ijms27177912 (PMC13566890; doi:10.3390/ijms27177912)
Supplement: Supplementary file 1 [file ijms-27-07912-s001.zip › Supplementary Material S2/novel_miR_62.pdf]

Provisional ID	:	novel_miR_62
Score total	:	0.7
Score for star read(s)	:	-1.3
Score for read counts	:	0
Score for mfe	:	0.4
Score for randfold	:	1.6
Score for cons. seed	:	
Total read count	:	1164
Mature read count	:	1129
Loop read count	:	35
Star read count	:	0

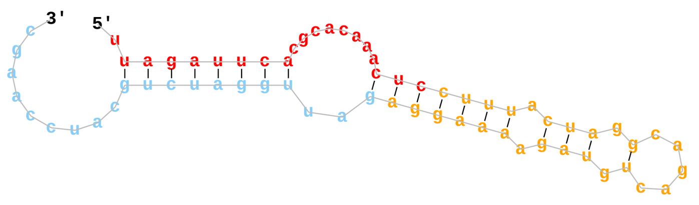

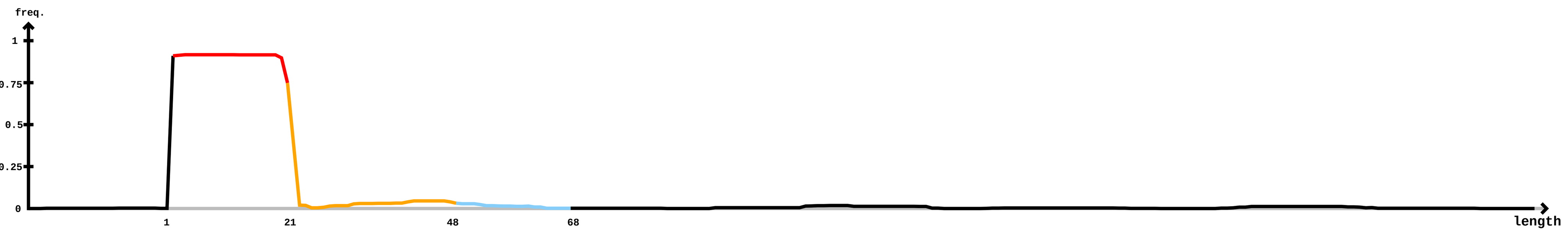

Mature Star

5'- uacuuguggacacaaaaacccauuagauucacgcacaaacuccuuuacuaggcagacuguagaaaaggagauuggaucugcauccaagcguuuuugacagaaaguaacaaucuucuucaccuucgcacaaagaugaagaagaugacggugccaugcuauggccugucaaacaaaaaacauaucauucuccaggucagcaugcaauuugaagaaccucucauccauugaagcugcugucgacagagaugau -3' exp

Structure / reads	reads	mm	sample
(((((...(...(((((((.(.((.(((.((....(((.(((((((.(((.(.....).))).))))))).)))....)).))).))).))))))).)...)))))....(((((((((.(((......))).)))))))))(((((.(((((...)))))))))))...........(((((.(((...(.(((((..((((.(((........)))...))))....))))).)....))))))))	reads	mm	sample
.Gcuuguggacacaaaaacc	2	1	LT-3
.............aaaaacccauuagauucacU	1	1	JCK-2
......................uuagauucacgcacaaac	2	0	JCK-2
......................uuagauucacgcacaaac	9	0	LCK-3
......................uuagauucacgcacaaac	1	0	JCK-1
......................uuagauucacgcacaaac	5	0	LT-3
......................uuagauucacgcacaaac	5	0	LT-1
......................uuagauucacgcacaaacu	4	0	JCK-1
......................uuagauucacgcacaaaUu	5	1	LCK-3
......................uuagauucacgcacaaacu	24	0	LT-3
......................uuagauucacgcacaaacu	33	0	LT-1
......................uuagauucacgcacaaacu	31	0	LCK-3
......................uuNgauucacgcacaaacu	1	1	LT-2
......................uuagauucacgcacaaacu	21	0	LCK-1
......................uuagauucacgcacaaacu	14	0	JCK-3
......................uuagauucacgcacaaacu	23	0	LCK-2
......................uuagauucacgcacaaacu	22	0	LT-2
......................uuagauucacgcacaaacu	5	0	JCK-2
......................uuagauucacgcacaaacuU	5	1	LT-3
......................uuagauucacgcacaaacuc	4	0	JT-3
......................uuagauucacgcacaaacuU	2	1	LT-2
......................uuCgauucacgcacaaacuc	1	1	LCK-3
......................uuagauucacgcacaaacuU	7	1	LCK-3
......................uuagauucacgcacaaacuc	162	0	LT-2
......................uuagauucacgcacaaacuc	1	0	JT-2
......................uuagauucacgcacaaacuc	142	0	LCK-3
......................uuagauucacgcacaaacuc	104	0	LT-1
......................uuagauucacgcacaaacuc	33	0	LCK-1
......................uuagCuucacgcacaaacuc	1	1	LT-2
......................uuagauucacgcacaaacuc	27	0	JCK-1
......................uuagauucacgcCcaaacuc	1	1	LT-3
......................uuagCuucacgcacaaacuc	1	1	LT-1
......................uuagCuucacgcacaaacuc	1	1	LT-3
......................uuagauucacgcacaaacuc	37	0	LCK-2

Mature **Star**

uacuuguggacacaaaaaccca**uuagauucacgcacaaacuc**cuuuacuaggcagacuguagaaaagga*gauuggaucugcauccaagc*guuuuugacagaaaguaacaaucuucuucaccuucgcacaaagaugaagaagaugacggugccaugcuauggccugucaaacaaaaaacauaucauucuccaggucagcaugcaauuugaagaaccucucauccauugaagcugcugucgacagagaugau

uuagauucacgcacaaacuc	156	0	LT-3
uuagauucacgcacaaacuc	57	0	JT-1
uuagauucacgcacaaacuc	31	0	JCK-2
uuagauucCcgcacaaacuc	1	1	LT-1
uuagauucacgcacaaacuc	19	0	JCK-3
uuagauucacgcacaaacuU	2	1	LT-1
uuCgauucacgcacaaacuc	1	1	LT-1
uuagauucacgcacaaacuUc	1	1	JCK-3
uuagauucacgcacaaacucA	3	1	LT-1
uuagauucacgcacaaacucc	1	0	JCK-1
uuagauucacgcacaaacucU	14	1	LT-3
uuagauucacgcacaaacucU	11	1	LT-1
uuagauucacgcacaaacuUc	2	1	LT-2
uuagauucacgcacaaacucA	1	1	JCK-3
uuagauucacgcacaaacucc	1	0	LT-3
uuagauucacgcacaaacucU	8	1	JCK-1
uuagauucacgcacaaacucU	6	1	JCK-2
uuagauucacgcacaaacuUc	3	1	LT-1
uuagauucacgcacaaacucU	26	1	LT-2
uuagauucacgcacaaacucN	1	1	JCK-1
uuagauucacgcacaaacuUc	1	1	JCK-2
uuagauucacgcacaaacucU	8	1	JCK-3
uuagauucacgcacaaacucU	7	1	LCK-3
uuagauucacgcacaaacucA	1	1	LCK-3
uuagauucacgcacaaacucUu	1	1	LCK-3
uuagauucacgcacaaacucUu	1	1	LT-3
uuagauucacgcacaaacucUuu	6	1	LT-3
uuagauucacgcacaaacucUuu	1	1	LT-1
uuagauucacgcacaaacucGuu	2	1	LT-1
uuagauucacgcacaaacucGuu	9	1	JT-1
uuagauucacgcacaaacucUuuu	4	1	JCK-1
uuagauucacgcacaaacucUuuu	1	1	JCK-3
uagauucacgcacaaacu	1	0	LT-2
uagauucacgcacaaacuc	1	0	LCK-3
uagauucacgcacaaacuc	2	0	LT-3
agauucacgcacaaacuc	2	0	JCK-3
agauucacgcacaaacuc	2	0	JCK-1
acuaggcagacuguagaaaagg	4	0	LT-1
acuaggcagacuguagaaaagga	1	0	LT-3
cuaggcagacuguagaaaagg	1	0	JCK-1
cuaggcagacuguagaaaagg	2	0	LT-2
cuaggcagacuguagaaaaggag	1	0	JCK-2
uaggcagacuguagaaaagga	6	0	JT-3
uaggcagacuguagaaaagga	3	0	JT-2
aggcagacuguagaaaaggag	1	0	JCK-1
aggcagacuguagaaaaggag	1	0	LT-3
aggcagacuguagaaaaggag	1	0	JCK-2
cagacuguagaaaaggagauu	6	0	LCK-3
cagacuguagaaaaggagauug	2	0	JCK-1
cagacuguagaaaaggagauug	3	0	JCK-2
cagacuguagaaaaggagauugga	2	0	JCK-1
agacuguagaaaaggagauug	2	0	JCK-1
agacuguagaaaaggagauug	1	0	JCK-2
cuguagaaaaggagauuggaC	1	1	JCK-2
uagaaaaggagauuggaucG	2	1	LT-2
gaaaaggagauuggaucugca	6	0	LCK-3
gaaaaggagauuggaucugca	1	0	LT-1
gaaaaggagauuggaucugcauc	1	0	JCK-2
gaaaaggagauuggaucugcauc	1	0	JCK-3
aaaaggagauuggaucugcauc	2	0	LT-1
aaaaggagauuggaucugcauc	5	0	LCK-3
auccaagcguuuuugacagaaag	2	0	JCK-1
uucuucaccuucgcacaaagaug	6	0	LCK-3
caaagaugaagaagaugacgg	3	0	JCK-2
caaagaugaagaagaugacgg	1	0	LT-3
caaagaugaagaagaugacgg	3	0	LCK-3
caaagaugaagaagaugacgg	5	0	JCK-3
aaagaugaagaagaugac	1	0	JCK-3
aagaugaagaagaugacggug	2	0	JCK-3
gaugaagaagaugacUgug	1	1	LT-1

Mature **Star**

```
uacuuguggacacaaaaacccauuagauucacgcacaaacuccuuuacuaggcagacuguagaaaaggagauuggaucugcauccaagcguuuuugacagaaaguaacaaucuucuucaccuucgcacaaagaugaagaagaugacggugccaugcuauggccugucaaacaaaaaacauaucauucuccaggucagcaugcaauuugaagaaccucucauccauugaagcugcugucgacagagaugau
.............................................................................................................................................................auggccugucaaacaaaaaaca.......................................................................   1   0   LT-3
..............................................................................................................................................................uggccugucaCacaaaaaacaua.....................................................................   1   1   LT-3
..............................................................................................................................................................uggccugucaaacaaaaaacaua.....................................................................   1   0   LT-2
................................................................................................................................................................gccugucaaacaaaaaacauaucauu................................................................   1   0   LT-1
....................................................................................................................................................................................................gcaugcaauuugaagaaccuc.................................   1   0   LT-1
....................................................................................................................................................................................................gcaugcaauuugaagaaccuc.................................   1   0   JCK-1
....................................................................................................................................................................................................gcaugcaauuugaagaaccuc.................................   1   0   JCK-2
......................................................................................................................................................................................................augcaauuugaagaaccucuc...............................   1   0   JCK-3
......................................................................................................................................................................................................augcaauuugaagaaccucuc...............................   1   0   JCK-1
.......................................................................................................................................................................................................ugcaauuugaagaaccucuca..............................   1   0   JCK-2
.......................................................................................................................................................................................................ugcaauuugaagaaccucuca..............................   2   0   LT-1
.......................................................................................................................................................................................................ugcaauuugaagaaccucuca..............................   1   0   JCK-1
.......................................................................................................................................................................................................ugcaauuugaagaaccucuca..............................   1   0   JCK-3
.........................................................................................................................................................................................................caauuugaagaaccucucauc............................   1   0   JCK-1
.........................................................................................................................................................................................................caauuugaagaaccucucauc............................   1   0   JCK-2
.........................................................................................................................................................................................................caauuugaagaaccucucauc............................   1   0   JCK-3
.........................................................................................................................................................................................................caauuugaagaaccucucauc............................   2   0   LT-3
.............................................................................................................................................................................................................................ccauugaagcugcugucg...........   1   0   JCK-2
.............................................................................................................................................................................................................................ccauugaagcugcugucg...........   1   0   JCK-1
```
